# Growth of broken crystals tracked in 4D using X-ray computed tomography and its influence on impurity incorporation

**DOI:** 10.1038/s41598-024-73127-y

**Published:** 2024-09-23

**Authors:** S. A. Schiele, T. Haider, H. Briesen

**Affiliations:** Chair of Process Systems Engineering, Technical University of Munich, Gregor-Mendel-Str. 4, 85354 Freising, Germany

**Keywords:** Chemical engineering, Chemical engineering, Thermodynamics, Imaging studies, Process chemistry

## Abstract

Crystallization is a commonly used unit operation for separation and purification. During processing, crystals may break due to mechanical stress, e.g., intentionally by milling or unintentionally through collision with stirrers. This study investigates the growth of broken crystals in three dimensions using X-ray micro-computed tomography. The results show that damaged regions of crystals grow faster than faceted regions, and crystals become faceted through growth. Initially, this happens on a microscale, producing faceted but concave regions on the crystal surface. Eventually, crystals become convex. Shape-healing through growth incorporates inclusions in the crystals. These findings have important implications for designing and optimizing crystallization processes in the pharmaceutical, food, and chemical industries, as purity is often a critical quality criterion adversely affected by inclusions. In addition, the kinetics in crystallization processes are likely to be strongly affected by the growth of non-faceted and concave crystals.

## Introduction

Suspension crystallization is a unit operation for separation, formulation, and purification^1,2^. Crystals are suspended in a supersaturated solution and grow. Crystal growth occurs when dissolved solute molecules attach to crystals in alignment with a material-specific lattice. In theory, alignment according to the lattice excludes other molecules, thereby achieving purification. The lattice causes isotropic surface energies. Crystal growth produces faceted crystals according to planes of minimal energy in such lattices. Faceted growth is why the predominantly applied crystal growth model assumes displacement of crystal faces in their normal direction^3–5^. Such shapes can be achieved under well-controlled reaction conditions.

However, slurries in crystallization equipment are agitated and pumped for improved heat and mass transport. This practice can unintentionally lead to damaged crystals. In some applications, crystals are intentionally damaged. For example, milling controls the size of seed crystals^6–8^ and product crystals^9–13^ or generates nuclei in continuous crystallization^8,14^. Therefore, crystals do not always have perfect facets in practice. Damage may occur through abrasion or attrition^15,16^. As a result, crystals become round^17^. Another phenomenon is breakage^18,19^, where impact shatters crystals into pieces, resulting in non-faceted, potentially concave crystal regions. The established face displacement growth model does not apply to damaged crystals because faces defined by the crystal lattice neither describe round nor concave crystals.

In previous work, we observed the growth of abraded, round potash alum (PA) crystals through X-ray micro-computed tomography (µCT) in single crystal growth experiments^20^. We used the resulting 3D images to parametrize a growth model that describes non-faceted convex crystals’ size and shape evolution. We observed shape healing: damaged regions of crystals grow fast to become faceted. Process simulations revealed that the growth of damaged crystals significantly affects the apparent mass crystallization rate in processes^21^.

Breakage leads to more complex shapes (i.e., concave) than abrasion. There is little literature on the growth of non-faceted concave crystals. Nguyen et al.^22^ used µCT to investigate the growth of crystal agglomerates. The crystals were concave but faceted. In that study, agglomerates consisted of faceted primary crystals, and the authors observed how faceted growth of the primary crystals leads to the inclusion of impurities in the agglomerates. In the context of the growth of broken crystals, fragments grow at increased rates in terms of an increase in overall mass^23^. Traditionally, such effects are described as size-dependent growth^24–28^ or growth rate dispersion^23,28–31^. We discussed before^21^ that both approaches are non-mechanistic descriptions and may lead to misinterpretation of experimental data. An in-silico approach^32^ describes the growth of broken seed crystals by fast-growing faces that do not occur in a non-damaged crystal. However, the rates were not experimentally determined, and whether broken regions grow according to faceted growth theories is unclear. In a recent study, the growth of two broken paracetamol crystals was analyzed experimentally by a series of 2D images^33^. The results show that growth in the fractured region is fast and leads to shape healing. However, the authors describe the growth of the broken region as face displacement.

We find the description of broken crystals through convex faceted shapes or even simpler shape models—as currently applied in the literature—unsatisfactory. In this study, we observe the growth of two broken aluminum potassium sulfate (potash alum, PA) crystals in three dimensions over time (4D). Here, it is not the goal to treat many crystals and to get statistically relevant information. Instead, we observe highly shape-dependent growth and focus on two crystals in the greatest possible detail.

## Materials and methods

We obtained ethanol (≥ 99.8%) and aluminum potassium sulfate (≥ 99% Ph. Eur.) from Carl Roth GmbH & Co. KG, and we used deionized water for all experiments.

### Growth experiment

Our growth experiment aims to track single, broken PA crystal fragments in 4D. The growth experiment is according to the protocol developed in our previous work^20^. In brief, glue (UHU Sekundenkleber Gel, UHU GmbH & Co. KG, Germany) fixes a crystal fragment (i.e., seed) to a sample holder. Micro-CT yields a 3D image of the seed in its initial state. Subsequently, the seed is grown in a clear supersaturated solution (relative supersaturation σ = 0.0174) at 20 ℃ for time steps specified in the results section. Then, we wash the crystal using an anti-solvent (50% (m/m) ethanol/water), dry it, and image it again. We repeat this cycle until the fragments appear fully faceted. The procedure yields a series of 3D images of the same crystals at different stages of their growth. The relevant differences to our previous work are (A) that we here use concave fragments as seeds instead of convex abraded round seeds, (B) we chose a higher resolution for µCT imaging (section "µCT imaging") to visualize concavities properly, and (C) we were only able to include one crystal per µCT scan due to the demand for higher resolution.

Note that measuring submerged crystals is not possible using our imaging setup due to the bad contrast between solution and crystal and lots of blur, making the intermitted washing and drying necessary. Water (i.e., solvent) is a strong absorbent to X-Ray radiation, and the solution contains strongly absorbing metal salts (i.e., solute) as well. To verify that the observed phenomena are not just an artifact of the washing and or drying, six fragments are suspended in a supersaturated solution and left there with gentle agitation, moving them at the bottom of the crystallization vessel. Images of the final shape are shown in section “Results” of the supporting material together with more details on this experiment.

### Seed production

We produce PA seed crystals by placing saturated PA solution (25 ℃) in a Petry dish covered with perforated parafilm. Saturation concentration is calculated according to Kovačević et al.^34^. Natural evaporation drives crystallization. Crystals are harvested once they reach a size of approximately five millimeters after a few days. Finally, crushing a crystal using a spatula produces seeds for the growth experiments. Fragments are selected based on size (~ 500 µm) and shape. We aimed for fragments with no crystal facets matching the material-specific Miller indices ([100], [110], [211], and [111]); other than that, the selection was arbitrary.

### µCT Imaging

Custom software (Matrix Technology, Germany) based on CERA software (Siemens AG, Germany) constructs the 3D images from 2000 radiographic 2D projections. We acquire the projections using a custom μCT system (XCT-1600HR; Matrix Technology AG, Germany). The voxel spacing for the images is 2 μm. Each 3D image visualizes a volume of 4 × 4 × 2 mm^3^ (2000 × 2000 × 1225 voxel^3^). A µCT measurement at these settings takes roughly two hours.

### Image analysis

Image analysis aims to segment the crystals and sample holders from the µCT images, align images of different times, calculate volume and surface areas, segment inclusions in the crystals, and finally classify surface area elements according to the Miller indices. The following sections explain these points separately.

#### Segmentation

**Fig. 1:**
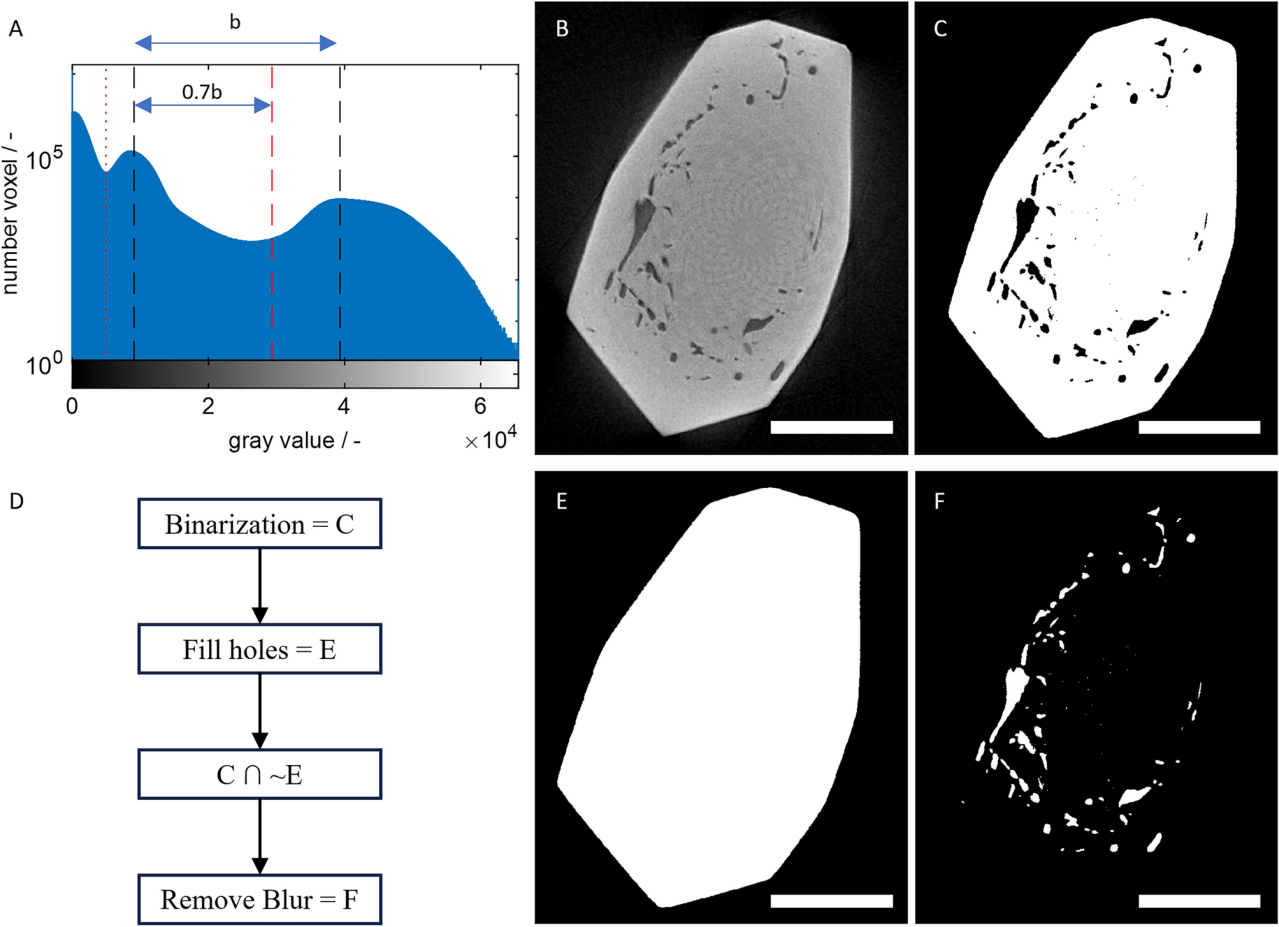
(**A**): Image histogram of the 57 min image of crystal 1, Otsu threshold (dotted red line), the peak of the sample holder (left dashed black line), the threshold for crystal segmentation (dashed red line), the peak of the crystal (right dashed black line); (**B**): Slice of the raw 3D image; (**C**): Slice of the segmented 3D crystal image; (**D**): summary of the basic image analysis protocol for inclusion segmentation; (**E**): C with filled holes; (**F**): Cut set of C and inverted E with blur removed; scale bars are 500 µm.

Figure 1A shows an exemplary gray value histogram of a raw µCT image. The first peak represents the background, the second is the sample holder, and the third is the crystal. Segmentation of the crystal is achieved by binarization between the second and third peaks. The threshold was set to a manually determined factor of 0.7 of the distance between the peaks (dashed red line in Fig. 1). The binarization using the threshold between the first and second peak (Otsu’s threshold, dotted red line in Fig. 1) and the threshold for crystal segmentation extracts the sample holder.

#### Alignment

Placing samples in the µCT in the same position is challenging. Therefore, an alignment procedure is necessary to overlay images of crystals at different stages of their growth. The alignment is based on our previous work^20^ but differs in some relevant aspects described in the supporting material. In brief, our program registers unchanged features in the images of the sample holders. It calculates a translation in 3D and rotation around the z-axis based on the features for all crystals to overlay well.

#### Volume and surface area

The volume of each crystal is calculated based on the number of voxels and the volume of a single voxel (i.e., 8 µm^3^). The first step in calculating surface area is to apply a Gaussian filter (Matlab’s *imgaussfilt3* function with a standard deviation of 3 voxels) to the binary volume images for smoothing. Subsequently, we triangulate the surface using Matlab’s *isosurface* function (isovalue is 0.5) and select the largest connected surface structure (using the function *splitFV* from Matlab’s file exchange^35^). The surface area is then the sum of all surface areas of all triangles.

#### Inclusion segmentation

In Fig. 1B, C, inclusions are visible as dark regions inside the bright crystal. These inclusions are extracted by segmenting the holes in the last image of each growth experiment. The procedure is sketched in Fig. 1D and explained in the following. The examples in Fig. 1 are in 2D for better visualization of the concept but are applied to 3D images for the results. The first step in segmenting the inclusions is filling the holes (Matlab’s *bwareaopen* function on the complemented image, Fig. 1E). The algorithm classifies all volume that is part of the filled image but not of the original image as inclusion. As a rule of thumb, an object with twice the length scale of a voxel (i.e., consisting of eight voxels) is detectable using our imaging setup. Therefore, and with a margin to reduce blur, inclusions smaller than ten voxels are considered noise and are removed (Matlab’s *bwareaopen* function, Fig. 1F). The result is a volume image with inclusions incorporated throughout the growth process. In the results section, the images of earlier time points show only those inclusions that are close (10 voxel distance) to the surface of the corresponding time point.

#### Surface element classification

The surfaces of crystals are triangulated (see above). The normal vector of each triangle is compared to a set of normal vectors defined by the Miller indices [111], [110], [100], and [211] that are known from the literature^20,36^ and cubic symmetry. Suppose the angle between the triangle’s normal vector and a vector defined by a Miller index is less than 7.5°. In that case, the triangle is associated with the corresponding Miller face group. For this assignment, it needs to be known how the crystal is orientated in the µCT image. The orientation is determined based on the final image of a growth experiment because then the crystal is fully faceted. Determining the orientation based on the final image is only possible because the crystal is glued to the sample holder, and the alignment procedure (section "µCT Imaging") ensures that the sample holder is orientated and positioned the same in each image. Three points on a [100] face define a normal vector ${{\varvec{n}}}_{100}^{*}$. The rotation matrix ${{\varvec{R}}}_{1}$ rotates ${{\varvec{n}}}_{100}^{*}$ to match $${{\varvec{n}}}_{100}={\left(\begin{array}{ccc}1& 0& 0\end{array}\right)}^{T}$$ such that ${{\varvec{n}}}_{100}^{*}\cdot {{\varvec{R}}}_{1}={{\varvec{n}}}_{100}$. This rotation is applied to all vertices of the surface. Then, for a second rotation, three points are sampled on a [110] plane, a normal vector ${{\varvec{n}}}_{110}^{*}$ to these points is calculated, and $${{\varvec{n}}}_{110}^{*}\cdot {{\varvec{R}}}_{2}={{\varvec{n}}}_{110}={\frac{1}{\surd 2}\left(\begin{array}{ccc}1& 1& 0\end{array}\right)}^{T}$$. Using the rotation ${\varvec{R}}={\left({{\varvec{R}}}_{2}{{\varvec{R}}}_{1}\right)}^{-1}$ the normal vectors defined by Miller indices can be rotated to match the µCT images. Which face belongs to which Miller index is determined using our previously published face detection algorithm^37,38^.

## Results

**Fig. 2:**
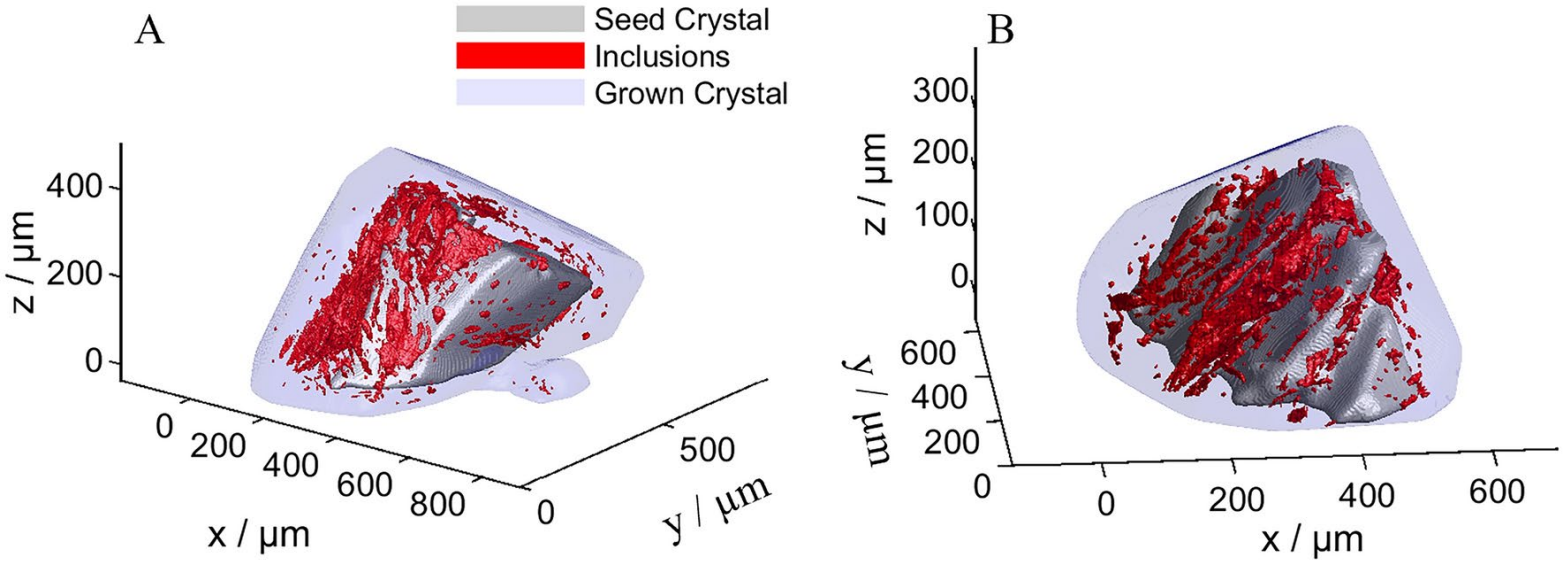
Crystal 1 (**A**) and 2 (**B**) showing the seed, detected inclusions, and the final shape after growth overlayed.

As a first qualitative result, Fig. 2 shows an overlay of the seed crystals 1 and 2, the corresponding shape after the last growth step, and the detected inclusions. A corresponding video for each crystal is provided in the supporting material to illustrate the 3D nature of the images.

**Fig. 3:**
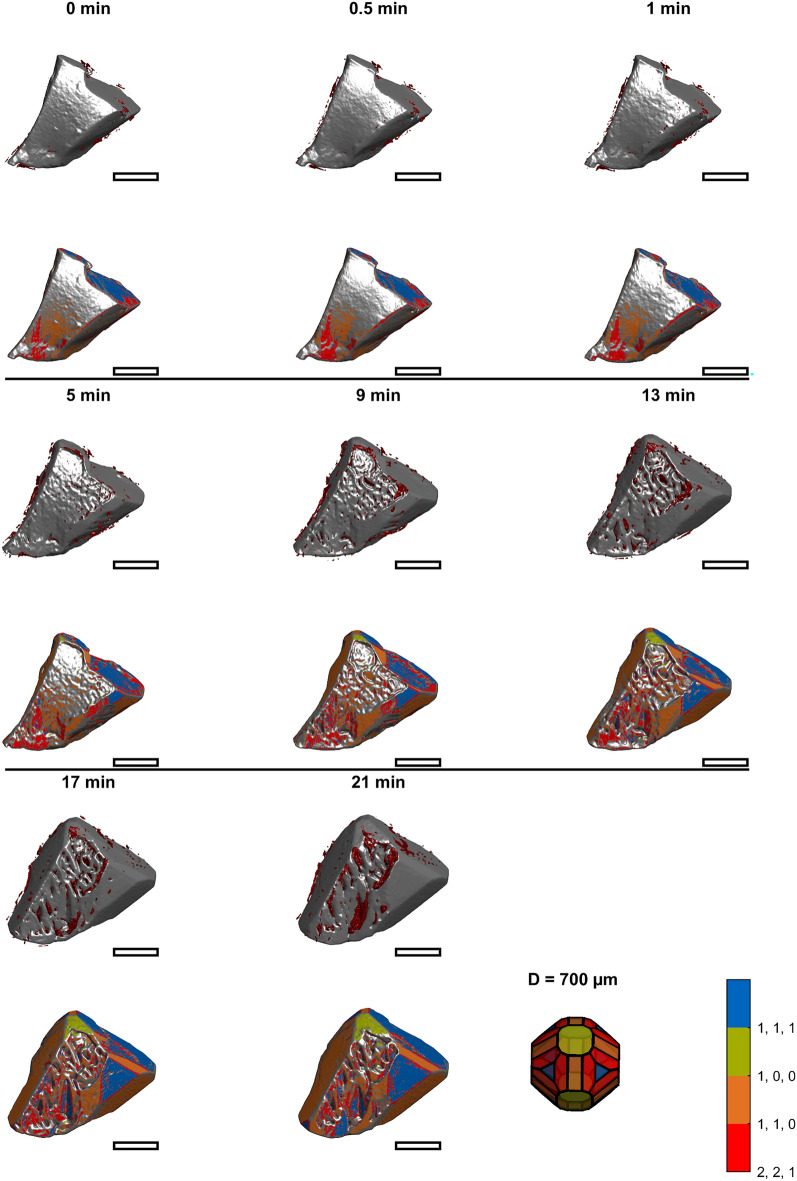
Rendered 3D images of crystal one at earlier times. The upper image of each time shows the raw image together with inclusions marked in red. The corresponding lower image visualizes the surface elements color-coded according to the Miller indices. The last tile shows a shape model of PA as a legend for the color coding and provides a scale. The diameter of its inscribing sphere is 0.7 mm. The scale bars approximate 0.35 mm.

**Fig. 4:**
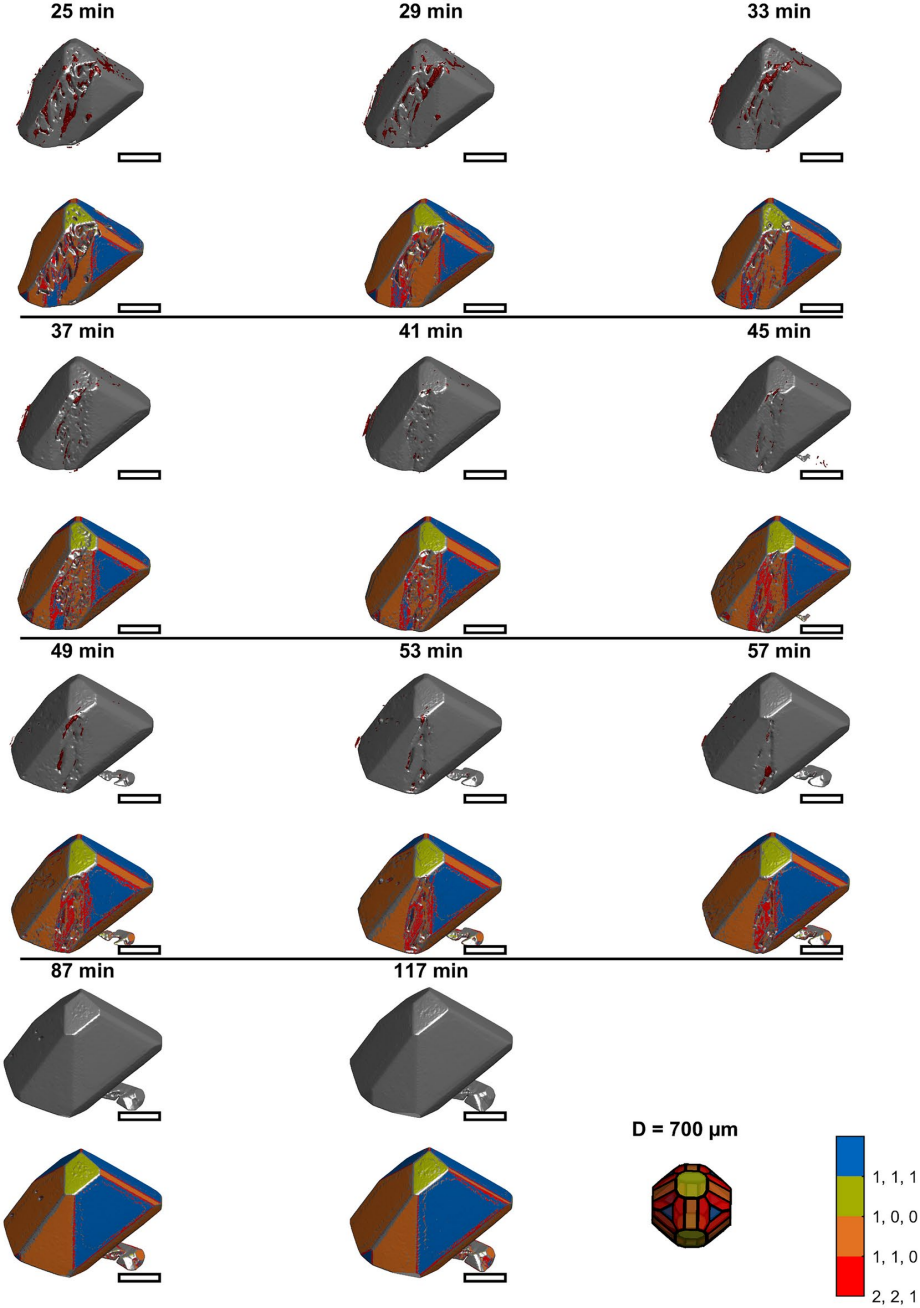
Rendered 3D images of crystal one at later times. The upper image of each time shows the raw image together with inclusions marked in red. The corresponding lower image visualizes the surface elements color-coded according to the Miller indices. The last tile shows a shape model of PA as a legend for the color coding and provides a scale. The diameter of its inscribing sphere is 0.7 mm. The scale bars approximate 0.35 mm.

**Fig. 5:**
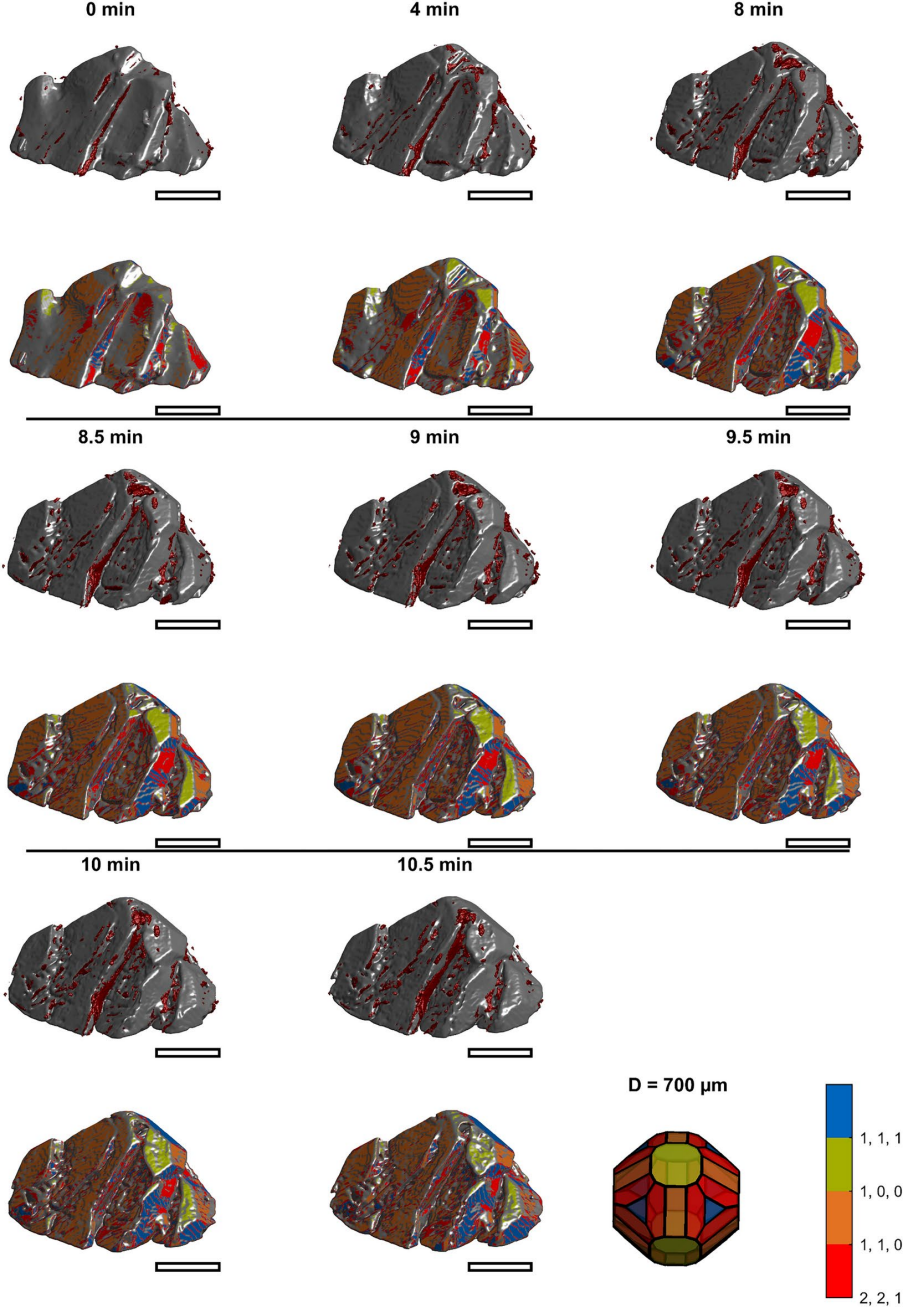
Rendered 3D images of crystal two at earlier times. The upper image of each time shows the raw image together with inclusions marked in red. The corresponding lower image visualizes the surface elements color-coded according to the Miller indices. The last tile shows a shape model of PA as a legend for the color coding and provides a scale. The diameter of its inscribing sphere is 0.7 mm. The scale bars approximate 0.35 mm.

**Fig. 6:**
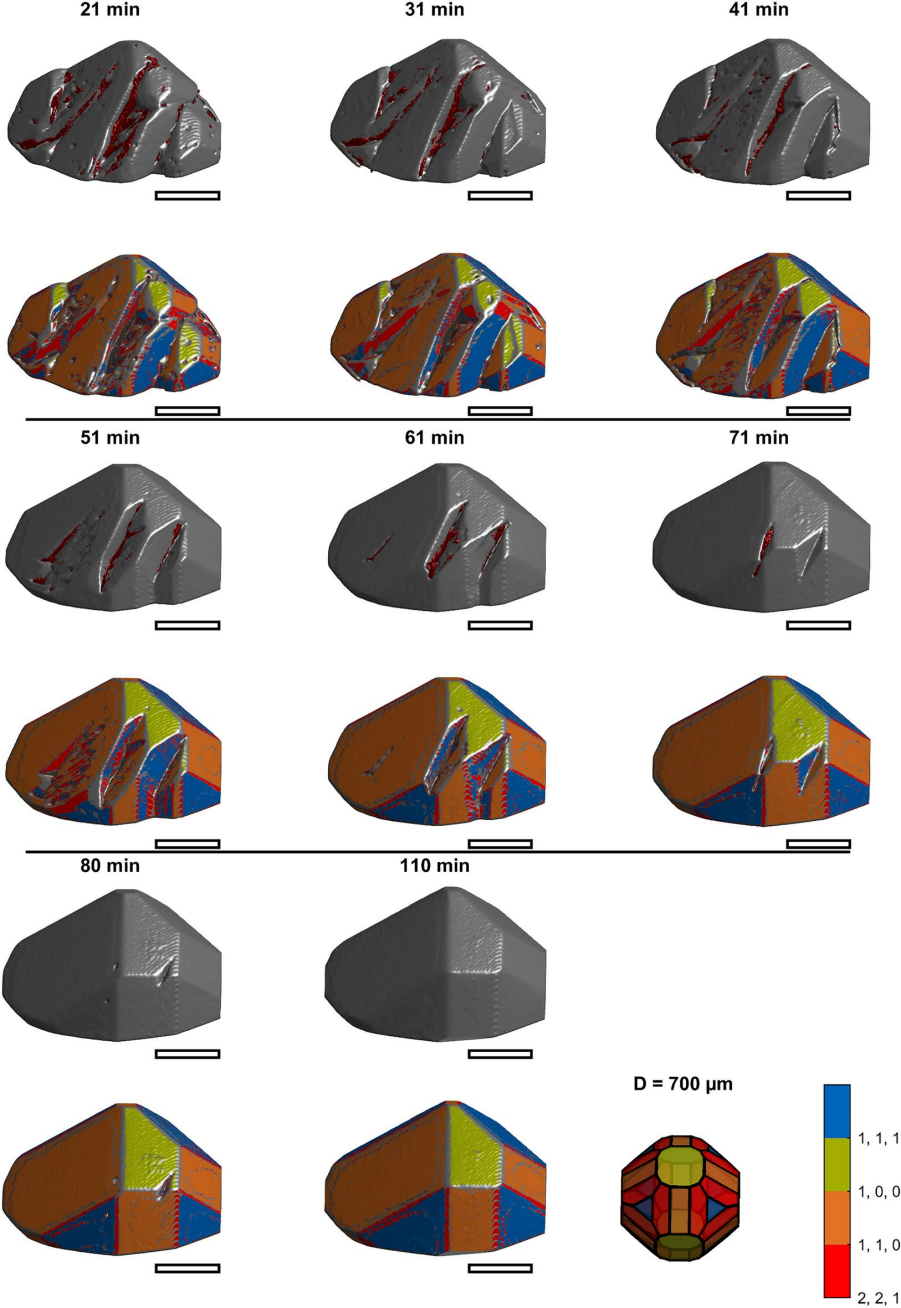
Rendered 3D images of crystal two at later times. The upper image of each time shows the raw image together with inclusions marked in red. The corresponding lower image visualizes the surface elements color-coded according to the Miller indices. The last tile shows a shape model of PA as a legend for the color coding and provides a scale. The diameter of its inscribing sphere is 0.7 mm. The scale bars approximate 0.35 mm.

Figure 3 through Fig. 4,5,6provide an overview of all images of the intermediate growth steps. Their raw STL files are provided online and can be viewed using standard software for viewing 3D Models (e.g., Microsoft 3D Builder)^39^. Larger images showing more details of selected time points are discussed in the following sections. Videos visualizing the corresponding growth are provided as supporting material. The upper images for each time display the volume classified as inclusion based on the final image (section "Inclusion segmentation", Fig. 2), which is close to the surface at the respective time in red. The corresponding lower images visualize the surface area in colors corresponding to Miller indices [111], [110], [100], and [211]. The crystal shape model in the bottom right of the figures acts as a color legend for the Miller indices and provides a scale. The images show that the initially concave seed crystals grow. The surface structure changes from initially relatively smooth to rough, and finally, both crystals are convex and faceted. Inclusions are predominantly detected where growth heals concavities. This is indicated in Fig. 3 through Fig. 6 by the red inclusions present where concavities occurred during growth. The following sections motivate studying shape-dependent growth and subsequently focus on describing three growth mechanisms identified from the images.

### Motivating shape-dependent growth

**Fig. 7:**
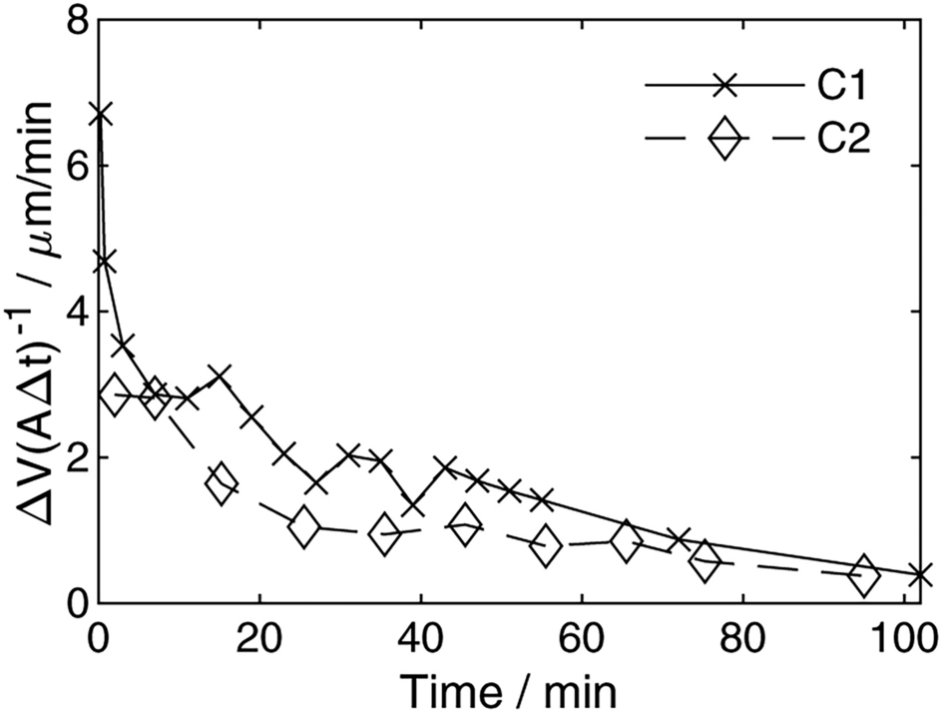
Averaged growth rate over time.

To evaluate overall growth, we determined the change in volume between the time points and divided it by the mean surface area of the corresponding times and the time step. This yields an averaged isotropic growth rate in terms of an increase in size (Fig. 7). Growth is fast (7 µm/min for C1 and 3 µm/min for C2) at the beginning of the experiments and decreases to below 1 µm/min towards the ends. We neither expect nor detect a stationary growth rate within the time scope of the experiment. Growth is highly shape-dependent^20^, and shape evolves drastically. Actual growth is anisotropic. In addition, inclusions are detected and make up a total of 2% of the grown crystal volume for both crystals. In the following sections, we will approach the anisotropic, shape-dependent growth behavior by focusing on regions with different growth rates.

### Mechanism 1: Rough growth

**Fig. 8:**
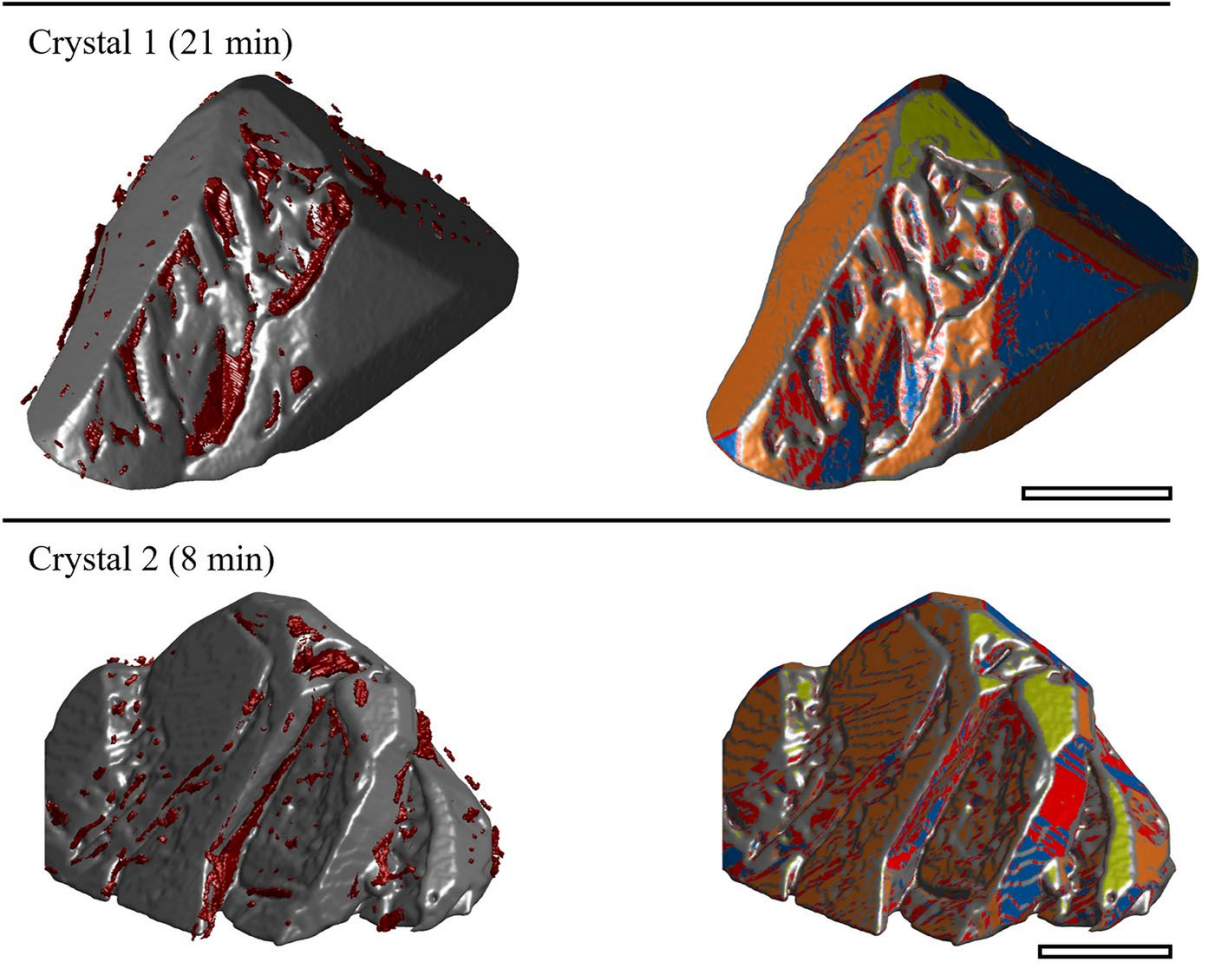
Rough growth at 21 min (top) of crystal 1, and at 8 min (bottom) of crystal 2. The scale bars approximate 0.35 mm.

The series in Fig. 3 shows that a rough region is formed within the first 5 min of the experiment for crystal 1. Crystal 2 (Fig. 5 and Fig. 6) has similar areas after 8 min. As growth continues, the rough regions become more pronounced. Between 5 and 25 min (crystal 1), the rough region forms what we hypothesize is a dendritic structure. Figure 8 shows magnifications of crystals 1 and 2 to show the rough regions with more detail (crystal 1: 21 min, Fig. 3; crystal 2: 8 min, Fig. 5). These images also illustrate that microscopic inclusions are incorporated in the valleys of the rough surface structure. It is further interesting that the rough surface has [110] (orange), [111] (blue), and [221] (red) faces.

### Mechanism 2: Healing of concavities

**Fig. 9:**
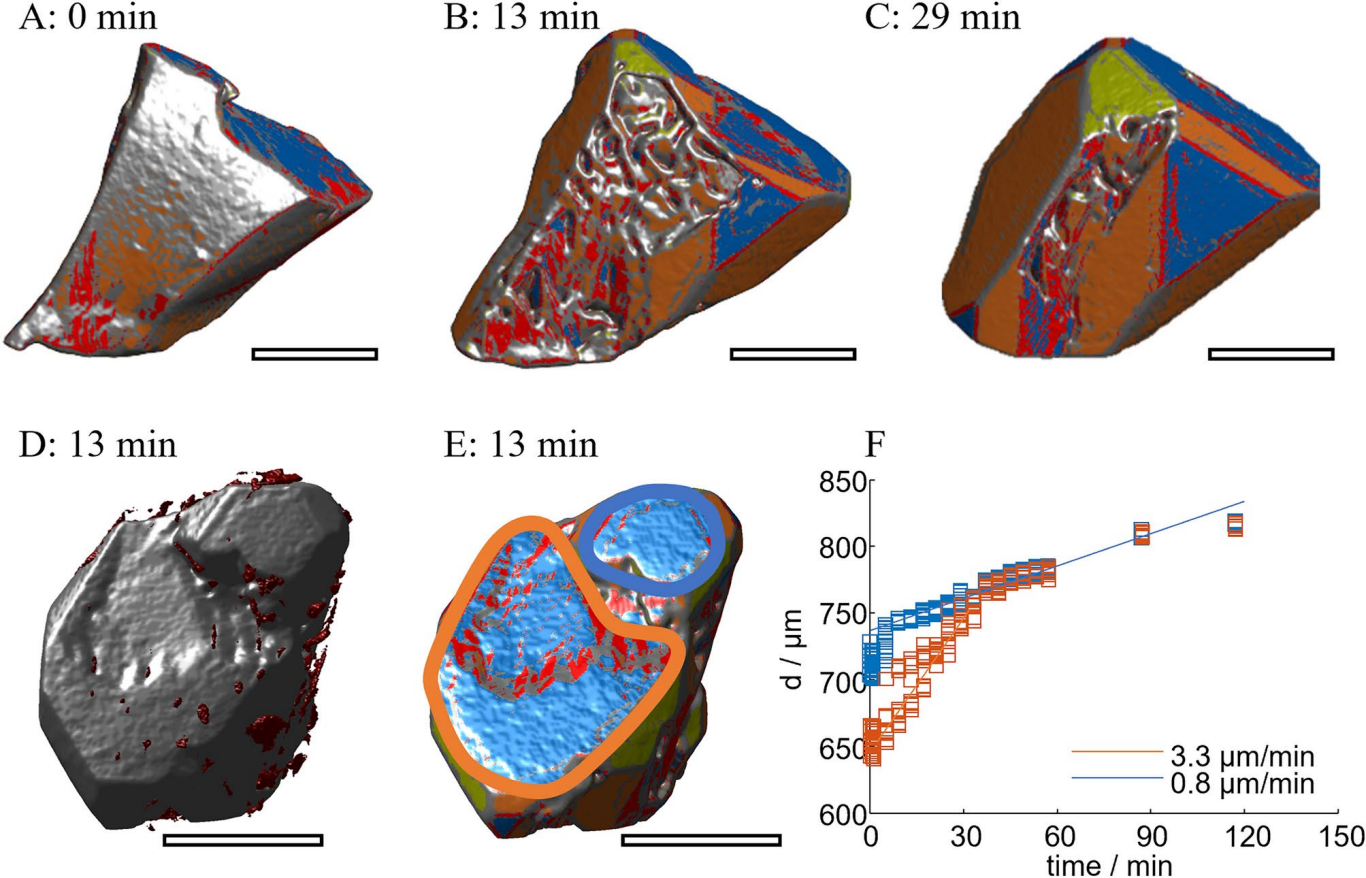
Growth of the top region of crystal 1. Images of the crystal show the crystal at (**A**): 0 min, (**B**): 13 min, (**C**): 29 min, and at 13 min from a downward perspective in (**D**) and (**E**). (**F**) shows the displacement in the normal direction of the [111] face (blue) of the orange and blue regions indicated in E. The scale bars approximate 0.35 mm.

Growth of the damaged regions yields a faceted but concave shape. For example, there are two parallel [111] faces at the seed crystal 1’s top (Fig. 4, focus on the region in Fig. 9). The larger, lower face catches up with the upper one at 29 min (Fig. 4A–C). Together, they form a single [111] face. Microscopic inclusions are detected at the boundary of the lower and upper parts (Fig. 9D). Analyzing the region more quantitatively, we manually sampled points on the upper face and separately on the lower face. Figure 9F displays the distances of the points to the origin in the direction of the corresponding [111] normal. This analysis reveals that growth in the lower region occurs at a rate of 3.3 µm/min until it catches up with the upper region. Both continue to grow at the same rate of 0.8 µm/min. Literature reports values between 0.6 and 2.2 µm/min^36,40–42^ for [111] faces of PA. In addition to growth in the direction normal to the [111] face, there are steps on the lower face forming [211] faces within the [111] face (Fig. 9B,D,E). The steps travel from the outer rim towards the step to the upper face.

**Fig. 10:**
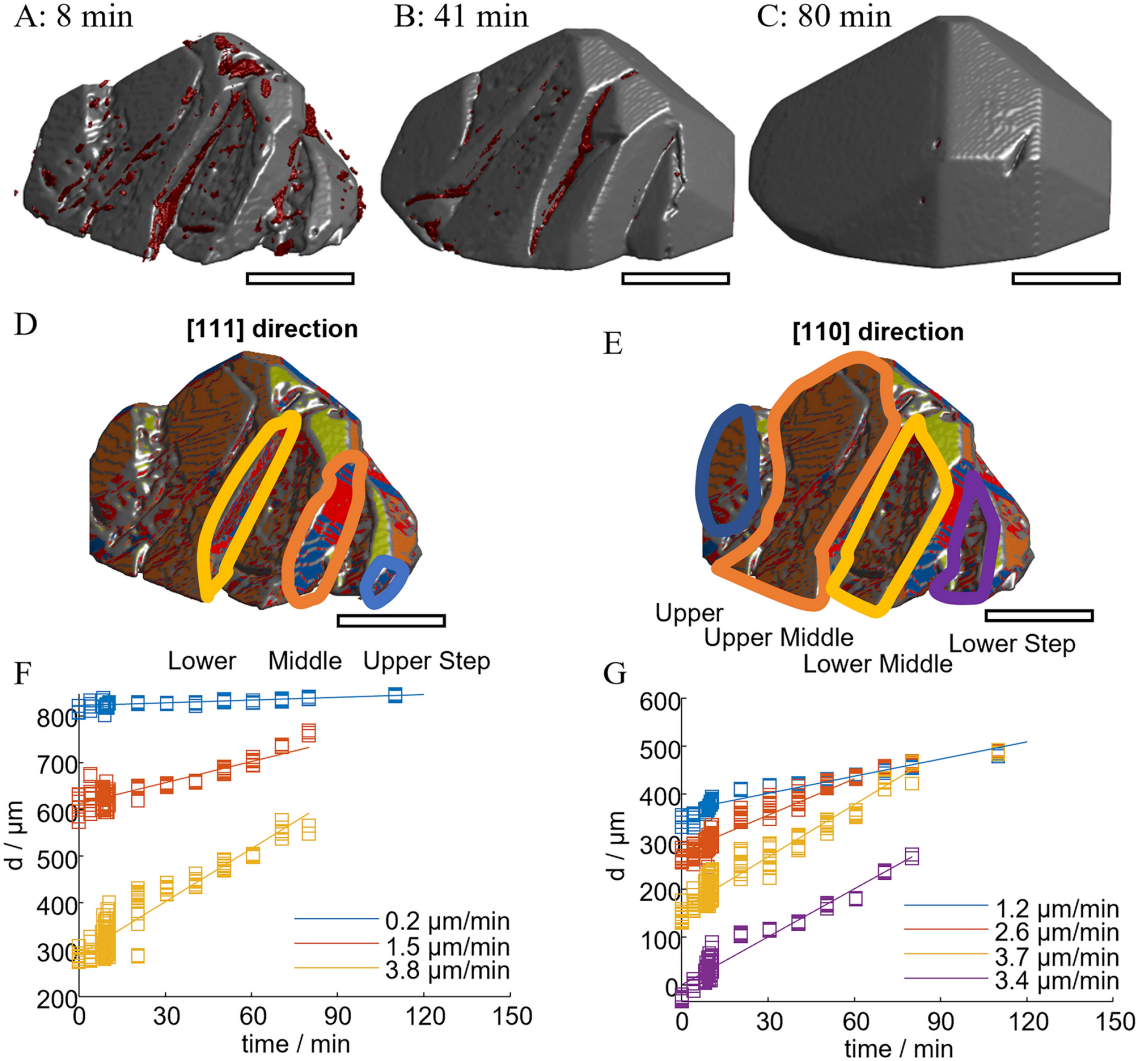
Growth of concave regions of crystal two. Images of the crystal show the crystals with inclusions at (**A**): 8 min, (**B**): 41 min, (**C**): 80 min and at indicating the studied regions in D and E. F and G show the displacement rates according to D and E, respectively. The scale bars approximate 0.35 mm.

A similar mechanism is observed for crystal 2 and illustrated in Fig. 10. The figure shows the 8, 41, and 80 min images (Fig. 10A–C) of the crystal with inclusions as an overview and two copies of the 8 min shape with surface orientation (Fig. 10D,E).

Figure 10D shows three regions of crystal 2 forming [111] faces marked by colored drawings. In Fig. 10F, these regions’ growth in the [111] direction is plotted in corresponding colors. The upper step grows at the slowest rate of 0.2 µm/min. The others grow at higher rates until they merge into other faces of the crystal at 80 min (c.f. Figure 10C). Notably, the increase in size in this direction is only affected by the growth of the upper step. Growth of the other steps affects the increase in volume until they disappear.

Figure 10E shows four regions of crystal 2 forming [110] faces by colored drawings. In Fig. 10G, these regions’ growth in the [110] direction is plotted in corresponding colors. Again, the upper step grows at the slowest rate (1.2 µm/min), limiting the increase in size in this direction. The other steps grow at increased rates, contributing to an increase in volume. The middle steps merge with the upper step at 60 and 80 min. The lower step merges into other faces at 80 min (c.f. Figure 10C).

### Mechanism 3: convex faceted growth

The fast growth of the lower steps and slow growth of the upper steps result in the healing of concavities and, finally, a convex shape. This shape continues to grow according to well-studied conventional faceted growth of convex crystals. Because the growth of faceted crystals is well-understood^43^, we do not study it here.

## Discussion

### Growth mechanisms

In crystallization, surface energy minima are planes that produce faceted crystals according to the unit cell. Unlike liquids or gases, molecules in crystals cannot rearrange to minimize surface energy, and surface energy minimization occurs through growth in a supersaturated environment. Here, surface energy minimization is observed to occur through three pathways:

- Formation of faceted concave rough regions (fastest)
- Concavities heal through fast growth
- Convex faceted crystals continue to minimize surface energy according to face-specific growth rates, eventually leading to octahedral crystals for this material system (slowest).

We did not find a detailed experimental description for the first two mechanisms in the literature. However, the literature predicts dendritic growth to result in solvent inclusions using phase field modeling^44–46^. The third mechanism is well studied in the literature ^3,4,36,43,47^ and hence not discussed here.

For the first mechanism, we hypothesize that it is most energetically favorable for molecules to attach where no plane of the unit cell is present (0 min in Fig. 3 and Fig. 5). Many steps, kinks, and alike will favor growth in damaged regions. Locally, fast growth produces a faceted but rough surface structure (Fig. 8). Overall, the crystals remain concave. We hypothesize that we observe dendritic growth where no crystal face is present, and growth is fast. Literature on dendritic growth is abundant for melt crystallization but not described for the growth of fragments in suspension crystallization. Generally, growth mechanisms are discussed on the grounds of supersaturation^43,48^. While supersaturation (not studied here) will undoubtedly have a substantial impact, we observe that the growth mechanism also depends on the crystal shape. At the same time, some regions grow slowly by face displacement, but damaged parts grow fast by rough growth.

For the second mechanism, we hypothesize that valleys of the dendrites and macroscopic steps seed molecular scale steps that lead to fast growth and, hence, filling of the valleys. This leads to fast growth of concavities and slow growth of faces that are part of the convex hull of a crystal. Even faces with the same Miller index grow at different rates. The slow growth of the convex hull limits the increase in size. The increase in volume is still fast because of the healing of the concavities. Growth rate dispersion (GRD)^28^ is traditionally employed to describe varying growth rates of crystals at the same thermodynamic conditions. GRD is attributed to surface structure^29,31^, defects^49^, or internal strain^30^. Here, faces orientated in the same direction grow at increased rates if they are part of a concavity but at a reduced rate once they merge into a face that is part of the convex hull. The steps cause shape-dependent accelerated growth in our crystals. In addition, we observe that, e.g., a convex [111] face of crystal 1 grows at 0.8 µm/min while one of crystal 2 grows at 0.2 µm/min. We cannot simply explain this by shape-dependent growth. However, keep in mind that even though both crystals are finally convex, they do have different shapes. In conclusion, we observe GRD in convex crystals and shape-dependent growth in concave crystals.

### Entrapment of Inclusions

Where concavities heal, we observe inclusions (c.f. Figure 10A,B). Different mechanisms^50,51^ may include impurities inside a crystal. One of the mechanisms is the encapsulation of solvent in the crystal^44,51^. Inclusions were also detected by Nguyen et al.^22^ using micro-computed X-Ray tomography. In that study, faceted growth of agglomerates entrapped solution. Here we find defects of sizes up to about 100 µm that make up 2% of the crystallized volume. Currently, our method allows for detecting inclusions through a different density and does not allow for determining what is inside the voids. Literature suggests it is a saturated solution^44,52^. We also observe inclusions in suspended damaged crystals (as opposed to fixed by the sample holder, see supporting material). Therefore, we are confident that neither the glue, the washing procedure, nor specific hydrodynamics caused by the fixation of the crystal close to the stirrer cause the inclusions. Surface energy minimization does not explain impurity inclusion. Instead, we hypothesize that local heat and mass transfer limitation leads to it. After concavities form to minimize local surface energy, valleys of the dendritic surface may be subject to diffusion limitation. The peaks are better supplied by fresh solution and grow faster. At the same time, crystallization releases energy of fusion, leading to slightly higher temperatures where flow is stagnant. In consequence, local supersaturation would be lower and growth slower. Once a peak merges with another, the solution can be entrapped in the valleys.

Similarly, some regions (crystal 1, Fig. 9) grow in opposing directions to close the gap between them. Inclusions are detected where two growth fronts meet. We hypothesize a similar mechanism where—once the opening is narrow—diffusion limitation in the valley hinders growth. Close to the outer surface, growth is supplied by fresh solution and closes the gap entrapping solution. In addition, where breakage leads to steps (crystal 2, Fig. 10), lattices may be deformed, leading to inclusions until growth healed the deformations. After the shape recovered, we did not observe inclusion for faceted growth.

### Relevance to processes

Our study focuses on two crystals that were tracked accurately in 4D. Even though the statistical relevance of the data is sparse, our findings have critical qualitative implications for process design, operation, and future research. Accurate growth description of broken crystals is relevant if, e.g., milling is applied to control the size and shape of suspended crystals^9–13^, seed crystals^6–8^, or used to control nucleation in continuous crystallization^8,14^.

#### Shape healing influences process kinetics

The literature largely ignores the effect of milling procedures—or the effect of damage in more general terms—on process kinetics. In most studies, measures characterizing convex shape models describe crystals. For example, a diameter describes what is assumed as a spherical particle in simple cases. Multiple size parameters can also define a polytope in more advanced methods. Because crystals are not faceted when damaged, we suggested a mechanistic process model that considers the particle volume, surface area, and a shape factor as disperse characteristics^21^. The concept of the model is that volume describes the size. The increase in volume depends on surface area and the fraction of surface area that grows slowly (faceted fraction described by shape factor) and another portion that grows fast because it is damaged. Simulations indicate that the growth of damaged convex crystals increases the mass crystallization rate significantly^21^. We expect a similar qualitative result for broken concave crystals. However, the parametrization of the model fails if crystals are concave because the underlying shape model assumes convex particles. In summary, the growth of broken crystals calls for more complex shape models that correctly couple volume and surface area evolution.

#### Impurity inclusion harms purification crystallization processes

We find that the healing of concavities incorporates 2 vol. % solution in macroscopic cavities. A way to remove such impurities is expensive recrystallization^53^. Further understanding of impurity inclusions may help to prevent impurity incorporation in the first place. Our study indicates that preventing breakage may help to reduce impurities. Teerakapibal et al.^54^ find that impurities are incorporated predominantly at the beginning of a seeded crystallization process. It is possible that the growth of damaged seed material led to inclusions there.

## Conclusion

We describe a method to track the growth of single crystal fragments in 4D. Initially, the fragments have a small portion of surface area orientated according to the Miller indices. The series of 3D images shows that the fragments of PA crystals undergo a shape transition through growth. Shape transition happens in three steps: (1) formation of faceted rough surface, (2) healing of concavities, and (3) classical faceted growth. The rough surface may be dendritic. The literature describes rough growth as when crystals grow fast in highly supersaturated environments. However, it is not related to occur dependent on shape. Our experiments were conducted at constant and relatively low supersaturation. The results indicate that faceted regions that are part of the convex hull of a crystal grow slowly by face displacement. Damaged regions form dendrites, grow fast, and form concave faceted regions. The concavities grow much faster (factor 2–10) than faces that are part of the convex hull and have the same Miller indices. Healing of concavities through growth incorporates impurities. Inclusions are harmful to purification crystallization processes. In addition, the kinetic involved in the shape healing process is complex. At the same time, shape healing affects volume growth rates and overall process behavior. Both kinetics and inclusions may be fundamental in practices where damaged crystals are present, e.g., when milling controls the size specification of a product or the seed crystals or nucleation in continuous crystallization (Supplementary videos).

## Supplementary Information

Supplementary Information 1.

Supplementary Video 1.

Supplementary Video 2.

Supplementary Video 3.

Supplementary Video 4.

## Data Availability

Raw data supporting the findings of this study are available from the corresponding author SAS on request. STL mesh files of the segmented crystals are provided online and can be viewed using standard software for viewing 3D Models (e.g., Microsoft 3D Builder)^39^.

## Abbreviations

µCT: Micro-computed X-ray tomography
2D: Two dimensional
3D: Three dimensional
4D: Four-dimensional
GRD: Growth rate dispersion
PA: Potash alum, aluminum potassium sulfate
