# Supplementary material for: Growth of broken crystals tracked in 4D using X-ray computed tomography and its influence on impurity incorporation
Source: Sci Rep. 2024 Sep 23;14:21999. doi: 10.1038/s41598-024-73127-y (PMC11424629; doi:10.1038/s41598-024-73127-y)
Supplement: Supplementary file 1 — Supplementary Information 1. [file 41598_2024_73127_MOESM1_ESM.pdf]

Supporting Material to

Growth of broken crystals tracked in 4D using X-Ray computed tomography and its influence on impurity incorporation

S.A. Schiele*, T. Haider, H. Briesen

Chair of Process Systems Engineering, Technical University of Munich, Gregor-Mendel-Str. 4, 85354, Freising, Germany

*Corresponding Author.

1 Video material

The following videos are available as separate files:

GrowthCrystal1.avi: Growth of crystal 1. The left shows the crystal and inclusions, and the right shows the orientation of the faces.

GrowthCrystal2.avi: Growth of crystal 2. See above

RotationInclusion1.avi: The initial shape of crystal one overlayed with its final shape and inclusions.

RotationInclusion2.avi: The initial shape of crystal two is overlayed with its final shape and inclusions.

2 STL Files

The STL files contain the surface mesh (i.e., 3D-Objects) of the crystals at each time point and can be viewed using standard software for viewing 3D Models (e.g., Microsoft 3D Builder). The length unit used in the files is mm.

The files are available at a separate data repository: www.doi.org/10.14459/2024mp1747486

3 Image Analysis

We place a sample in the µCT (initial measurement) for each growth cycle, move it to the reactor (growth), and place it in the µCT again. Putting the sample in the same position in the µCT for measurements is impossible. Consequently, the images are displaced in 3D (x, y, and z-direction) and rotated around the z-axis. The following section describes an algorithm that measures the displacement and the rotation and uses this information to correct the corresponding errors.

3.1 Segmentation

The first step is to segment the crystal and, separately, the sample holder. The main text of this publication explains the thresholds for segmentation. Sup. 1 shows two exemplary images of a sample holder and a crystal.

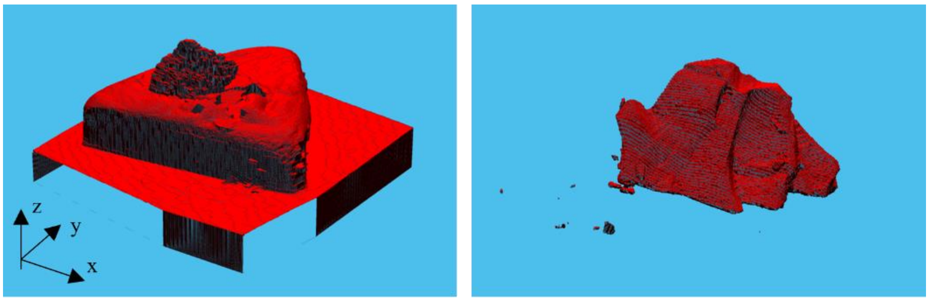

Sup. 1. Left: segmented sample holder. On the top of the sample holder, blur around the crystal is visible but well segmentable from the crystal and hence hollow. Right: segmented crystal

3.2 Registration

The sample holder is made from 3D-printed plastics and remains unchanged through crystallization. It has geometric features that classical image analysis techniques can easily detect. These features are

1. The number of voxels in each Z-plane defines the displacement in the Z-direction.
2. A triangular shape defines rotation and displacement in X/Y-direction.

3.2.1 Reference for Z-translation

Consider the the number of voxels per z-plane n for a reference in z-direction (orange line in Sup. 2, left) and then its derivative with respect to hight z (dn/dz, solid blue line in Sup. 2, left). The two peaks of dn/dz are identified using Malab’s *findpeaks* function. The center between the peaks defines a reference for displacement in the Z-direction (solid black line „center“ in Sup. 2, left; $z_{ref,i}$ for each time i, $i \geq 0$).

3.2.2 Reference for X/Y-translation

Cutting a slice out of the image of the sample holder between $z_{ref} - 30$ and $z_{ref} + 40$ (dashed lines in Sup. 2, left) segments the triangular shape (blue in Sup. 2, right). Projecting the 3D slice to a X/Y-plane (Matlab’s *any* function) generates a 2D binary image of the triangular shape. The center point of the triangle (Matlab’s *regionprops* function) provides references for displacement in X/Y-direction, respectively ($x_{ref,i}$ and $y_{ref,i}$). In summary, for displacement we write

$$\boldsymbol{p}_{ref,i} = (x_{ref,i} \quad y_{ref,i} \quad z_{ref,i})^T$$

$$\Delta \boldsymbol{p}_{ref,i} = \boldsymbol{p}_{ref,i} - \boldsymbol{p}_{ref,0}$$

Where vectors are in bold font and $\Delta \boldsymbol{p}_{ref,i}$ is a translational vector that moves the center point of each image to the center point of the first image.

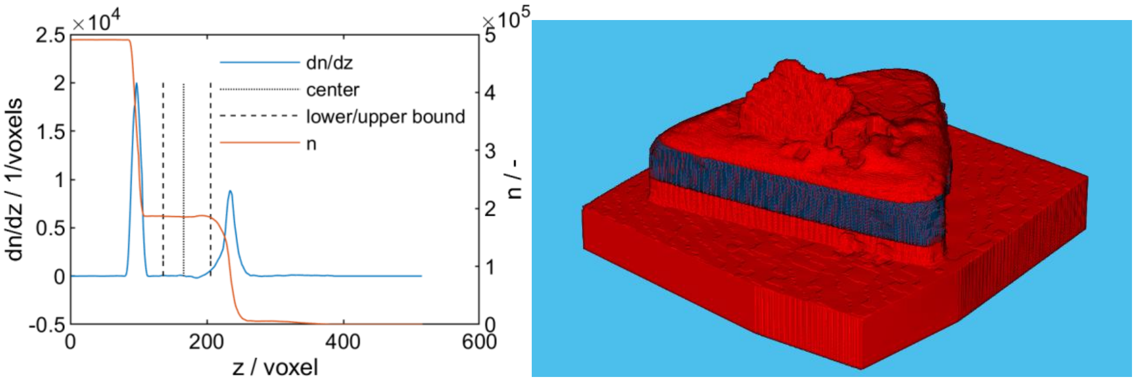

Sup. 2. Left: The number of voxels in each z-plane and corresponding derivative with respect to the z-hight. The dotted line marks the center between the peaks of dn/dz; the dashed lines mark the estimated boundaries of the triangle. Right: segmented triangle highlighted in blue.

3.2.3 Reference for rotation

The next step is to correct the rotation. The outline of the triangle is segmented by generating a gradient image, binarizing it, and dilating it by 5 pixels (Matlab's *imgradient*, *imbinarize,* and *imdilate* functions, Sup. 3). Three separate Hough transformations (Matlab's *hough* function) for the Hough angle ranges $-90° < \theta_1 \leq -30°$, $-30° < \theta_2 \leq 30°$, and $30° < \theta_3 \leq 90°$ parametrize one line per face of the triangle. The endpoints of the lines are extracted (Matlab's *houghpeaks* and *houghline* function), normal vectors to the lines are constructed, and the angles between each normal and the x-axis are calculated.

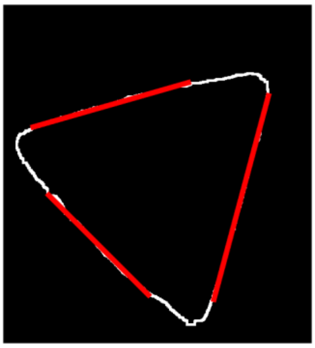

Sup. 3. 2D image of the triangle with lines identified by Hough transform.

This calculation yields a vector of angles $\boldsymbol{\alpha}_i$ for each time i. The correction of the rotation is found by solving the following minimization problem (Matlab's *fminsearch* function with $\Delta\alpha = 0$ as initial guess):

$$\Delta\alpha_i = \text{argmin}_{\Delta\alpha} \| f_\Delta(\boldsymbol{\alpha}_i, \boldsymbol{\alpha}_0, \Delta\alpha) \|_2$$

The function $f_\Delta(\boldsymbol{\alpha}_i, \boldsymbol{\alpha}_0, \Delta\alpha)$ calculates the difference between the two vectors but considers two challenges. 1) The entries in the vectors $\boldsymbol{\alpha}_i$ are in random order. 2) The angle between a normal and the X-axis is defined in the range $-90° < \alpha \leq 90°$. For illustration, consider a case where in two images each three lines were perfectly detected (i.e., no measurement inaccuracy and a perfect solution $\Delta\alpha_i$ with $\|f_\Delta\|_2 = 0$ exists): For the first image, the angles $\boldsymbol{\alpha}_0 = (-89° \quad -29° \quad 31°)^T$ were measured. For another image, $\boldsymbol{\alpha}_1 = (-32° \quad 28° \quad 88°)^T$. The obvious solution for the minimal rotation is that the second image needs to be rotated by 3°; However:

$$\boldsymbol{\alpha}_1 - \boldsymbol{\alpha}_0 + 3° = (-118 \quad 2 \quad 60)^T$$

The correct solution would consider that -89° and 88° are just 3° apart (i.e., almost parallel), and an appropriate algorithm would match the angles as follows

$$f_\Delta(\boldsymbol{\alpha}_1, \boldsymbol{\alpha}_0, \Delta\alpha = 3°) = \begin{pmatrix} 88° \\ 28° \\ -32° \end{pmatrix} - \begin{pmatrix} -89° \\ 31° \\ -29° \end{pmatrix} + 3° = \mathbf{0}$$

To achieve this, $f_\Delta(\boldsymbol{\alpha}_i, \boldsymbol{\alpha}_0, \Delta\alpha)$ is defined as

$$f_\Delta(\boldsymbol{\alpha}_i, \boldsymbol{\alpha}_0, \Delta\alpha) = \begin{pmatrix} \min\left(|\alpha^*_{i,1} - \alpha_{0,1}| \quad |\alpha^*_{i,2} - \alpha_{0,1}| \quad |\alpha^*_{i,3} - \alpha_{0,1}| \quad \left||\alpha^*_{i,1} - \alpha_{0,1}| - 180°\right| \quad \left||\alpha^*_{i,2} - \alpha_{0,1}| - 180°\right| \quad \left||\alpha^*_{i,3} - \alpha_{0,1}| - 180°\right|\right) \\ \min\left(|\alpha^*_{i,1} - \alpha_{0,2}| \quad |\alpha^*_{i,2} - \alpha_{0,2}| \quad |\alpha^*_{i,3} - \alpha_{0,2}| \quad \left||\alpha^*_{i,1} - \alpha_{0,2}| - 180°\right| \quad \left||\alpha^*_{i,2} - \alpha_{0,2}| - 180°\right| \quad \left||\alpha^*_{i,3} - \alpha_{0,2}| - 180°\right|\right) \\ \min\left(|\alpha^*_{i,1} - \alpha_{0,3}| \quad |\alpha^*_{i,2} - \alpha_{0,3}| \quad |\alpha^*_{i,3} - \alpha_{0,3}| \quad \left||\alpha^*_{i,1} - \alpha_{0,3}| - 180°\right| \quad \left||\alpha^*_{i,2} - \alpha_{0,3}| - 180°\right| \quad \left||\alpha^*_{i,3} - \alpha_{0,3}| - 180°\right|\right) \end{pmatrix}$$

Where $\boldsymbol{\alpha}^*_i = \boldsymbol{\alpha}_i + \Delta\alpha$. To apply this rotation, images are translated by $-\boldsymbol{p}_{ref,0}$, rotated by $\Delta\alpha_i$ around the z-axis and translated by $\boldsymbol{p}_{ref,0}$ (Matlab’s *imwarp* function).

4 Inclusions in suspended crystals

Six seed crystals were suspended in a supersaturated solution at the same conditions as in the growth experiments and left to crystallize for one hour. This experiment differs from the growth experiment in the main text because the crystals are not fixed to the sample holder but can float freely in a clear solution (only six crystals in the reactor). Due to their large size, they sedimented, but convection moved them around. They were harvested using a plastic Pasteur pipette and washed and dried on a Buchner funnel. Then, they were imaged using µCT, and slices of the images are shown below. The images indicate that also, for these crystals, inclusions are incorporated during growth. Even though there was no glue, intermediate washing, drying, and hydrodynamic conditions differed.

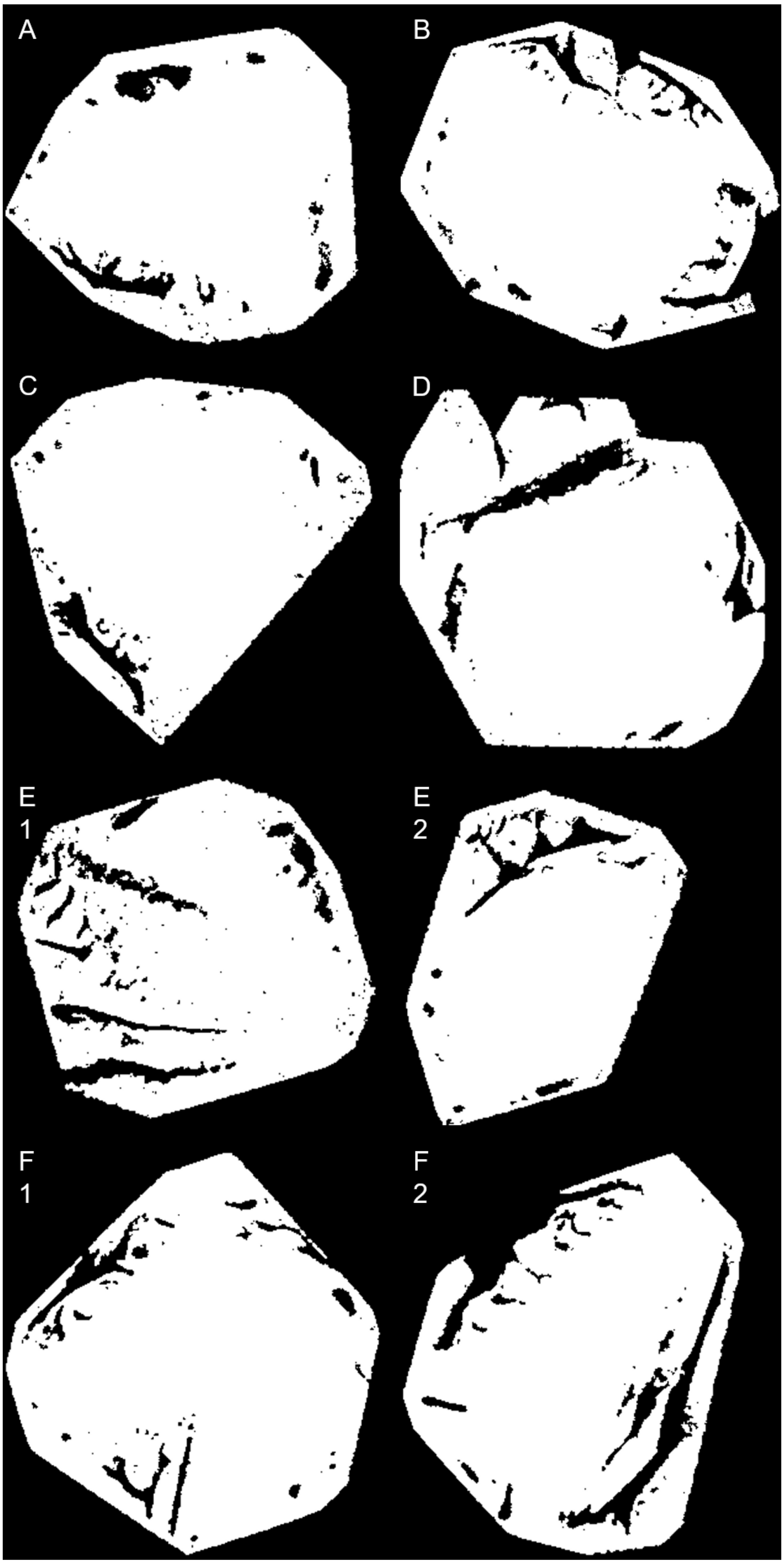

Sup. 4. Slices of six crystals (A-F) that grew without being fixed to a sample holder. The figure shows E and F from two perspectives (1 and 2). All crystals show similar inclusions as observed in the growth experiment discussed in the main text.
